# A Systematic Review of Effects of Physical Activity of Family Caregivers of Older Adults With Chronic Disease

**DOI:** 10.1093/geroni/igab046.1528

**Published:** 2021-12-17

**Authors:** Dawon Baik, Jiyoun Song, Aluem Tark, Heather Coats, Catherine Jankowski

**Affiliations:** 1 University of Colorado, Aurora, Colorado, United States; 2 Columbia University, Columbia University, New York, United States; 3 University of Iowa, University of Iowa, Iowa, United States; 4 University of Colorado, University of Colorado, Colorado, United States

## Abstract

More than 17 million family caregivers (FCGs) provide care for older adults with chronic illness in the US. Caregiving for older adults with chronic disease places a considerable burden on FCGs and they tend to neglect their personal health. Generally, physical activity (PA) programs benefit the physical and psychological health of FCGs. However, no review of PA randomized clinical trials (RCTs) focused on FCGs of older adults with chronic disease. In this systematic review, we analyzed the most recent trends (2010-2020) in RCTs identifying the effects of PA in this population. This review followed the Preferred Reporting Items for Systematic Reviews and Meta-Analyses guidelines. Electronic databases (PubMed, CINAHL, Embase, PsycInfo, Cochrane Library) were searched for publications dated from 2010 to 2020. All studies included were appraised for quality using the Cochrane Collaboration Risk of Bias Tool. Of the resulting 16 studies, most studies (n=11) targeted FCGs of older adults with dementia or cancer. Most FCGs were non-Hispanic white. PA interventions with mixed modes (e.g., aerobic and resistance exercise), mixed delivery methods (e.g., in-person and telephone) and mixed settings (e.g., supervised gym- and unsupervised home sessions) were used most frequently. PA interventions significantly improved psychological health but had inconsistent effects on physical health. Tailored PA programs, designed based on FCGs’ goals, preferences and limitations, may improve upon physical health outcomes. Future PA studies should include samples of racially and ethnically diverse FCGs of older adults representing a broader range of chronic diseases.

